# Similarities and Differences of Mental Health in Women and Men: A Systematic Review of Findings in Three Large German Cohorts

**DOI:** 10.3389/fpubh.2021.553071

**Published:** 2021-02-05

**Authors:** Daniëlle Otten, Ana N. Tibubos, Georg Schomerus, Elmar Brähler, Harald Binder, Johannes Kruse, Karl-Heinz Ladwig, Philipp S. Wild, Hans J. Grabe, Manfred E. Beutel

**Affiliations:** ^1^Department of Psychosomatic Medicine and Psychotherapy, University Medical Center, Johannes Gutenberg-University Mainz, Mainz, Germany; ^2^Department of Psychiatry and Psychotherapy, University Medicine Leipzig, Leipzig, Germany; ^3^Department of Psychiatry and Psychotherapy, University Medicine Greifswald, Greifswald, Germany; ^4^Faculty of Medicine and Medical Center, Institute of Medical Biometry and Statistics, University of Freiburg, Freiburg, Germany; ^5^Faculty of Mathematics and Physics, Freiburg Center of Data Analysis and Modelling, Mathematical Institute, University of Freiburg, Freiburg, Germany; ^6^Clinic for Psychosomatic Medicine and Psychotherapy, University Giessen and Marburg, Giessen, Germany; ^7^Department of Psychosomatic Medicine and Psychotherapy, Klinikum rechts der Isar, Technische Universität München, Munich, Germany; ^8^Preventive Cardiology and Preventive Medicine, Center for Cardiology, University Medical Center, Johannes Gutenberg-University Mainz, Mainz, Germany; ^9^Center for Thrombosis and Hemostasis, University Medical Center, Johannes Gutenberg-University Mainz, Mainz, Germany; ^10^DZHK (German Center for Cardiovascular Research), Partner Site Rhine-Main, Mainz, Germany

**Keywords:** systematic review, mental health, sex, gender, regional differences, Germany

## Abstract

In Germany, large, population-based cohort studies have been implemented in order to identify risk and protective factors for maintaining health across the life span. The purpose of this systematic review is to analyse findings from three large ongoing cohorts and to identify sex-specific prevalence rates, risk and protective factors for mental health. Published studies from the Cooperative Health Research in the Region Augsburg (KORA), the Study of Health in Pomerania (SHIP) and the Gutenberg Health Study (GHS)), representing the southern, north-eastern and middle parts of Germany, were identified through searches of the databases PubMed and Web of Science. A total of 52 articles was identified from the start of each cohort until June 2019. Articles reporting prevalence rates of mental health [*N* = 22], explanatory factors for mental health [*N* = 25], or both [*N* = 5] were identified. Consistent across cohorts, higher prevalence rates of internalizing disorders were found for women and more externalizing disorders for men. Risk and protective factors for mental health included social factors, lifestyle, physical health, body mass index (BMI), diabetes, genetic and biological factors. In all areas, differences and similarities were found between women and men. The most evident were the sex-specific risk profiles for depression with mostly external risk factors for men and internal risk factors for women. Gender was not assessed directly, therefore we examined whether socioeconomic and family-related factors reflecting gender roles or institutionalized gender could be used as a proxy for gender. Overall, this systematic review shows differences and similarities in prevalence rates and determinants of mental health indicators between women and men. They underline the importance of focussing on sex specific approaches in mental health research and in the development of prevention measures. Current research on mental health still lacks focus on gender aspects. Therefore, an increased focus on sex and gender in mental health research is of great importance.

## Introduction

Mental disorders have become major public health concerns affecting quality of life, work productivity and life expectancy (1) of a large proportion of the general population. Representative German studies have shown that approximately one in three women and one in four or five men had a diagnosis of a mental disorder in the previous 12 months (2). For most internalizing disorders (e.g., major depression and eating disorders), women are more frequently affected (3, 4), whereas for externalizing disorders (e.g., substance abuse) men are more frequently affected.

Mental health differences between women and men have been attributed to sex and gender differences. Rooted in genetics, anatomy, and physiology (5), sex represents a biological construct. In contrast, gender comprises psychosocial variables that differentiate women and men (6) elucidating societal conditions and offering explanatory models. Gender can be differentiated according to (1) gender roles: behavioral norms attributed to women and men in a given society; (2) gender identity: how people see themselves on the dimensions of femininity-masculinity; (3) gender relations: how individuals interact with or are treated by others based on ascribed or experienced gender; and 4) institutionalized gender: distribution of power between women and men in institutions in society which shapes social norms and justifies different expectations and opportunities for women and men (5). Sex differences in mental health can be explained by sex hormones (7, 8) and dysregulations in the hypothalamic-pituitary-adrenal (HPA) axis (7, 9), especially for stress-related psychiatric disorders. Gender differences in mental health can be explained by e.g., gender-based violence (10), low self-esteem (7) and belonging to a gender minority (11). The interplay between sex and gender is an important factor in mental health. Sex and gender interact in the development of diseases (12, 13) [e.g., depressive disorders (14)] and coping strategies (15). The association between biological sex and mental health in Europe is moderated by socioeconomic and family-related factors (16), which explain about 20% of the differences in mental health between women and men. Such factors are for example employment (16, 17), education, housekeeping or looking after children and income (16, 18). The inverse association between socioeconomic position and morbidity and mortality has been termed social gradient of health (19). While multiple inequalities between women and men (e.g., gender pay gap, lower pension due to maternity leaves and part-time employment) are known, interactions between sex and socioeconomic position, which may put women at a disadvantage have been understudied (19).

Sex and gender differences vary across countries and regions (20). In a study including 48 countries, males have consistently reported higher self-esteem than females, but the strength of this effect differed between countries (21). Furthermore, sex, gender, and cultural differences for self-reported emotional intensity (arousal) were found between the Chinese and German culture (22). However, in a study on the universality of emotions across 37 countries, no differences were found between women and men (23); in all countries men reported more powerful emotions (e.g., anger), whereas women tended to report powerless emotions (e.g., fear or sadness). A study focussing on similarities and differences in three psychological domains (cognitive domain, social and personality and well-being) combined numerous meta-analyses and identified only small differences between women and men (24). These differences remained consistent across countries. Regional differences regarding material living standards and employment conditions may also affect women and men differently (20). In examining mental health, it is therefore important to consider differences between countries and regions.

In Germany, large population-based cohort studies have been implemented in order to identify risk and protective factors for health in the general population. Major ongoing cohorts have been established between 1983 and 2007. These include the Cooperative Health Research in the Region Augsburg (KORA) [formerly Monitoring of Trends and Determinants of Cardiovascular Disease (MONICA)], the Study of Health in Pomerania (SHIP) and the Gutenberg Health Study (GHS), representing different German regions, respectively, the southern, north-eastern and middle part of Germany. KORA focusses on the fields of epidemiology, health economics, and health care research (25), SHIP contains a broad range of health and quality of life indicators (26) and GHS focusses on uncovering risk factors for several conditions, such as cardiovascular and mental diseases (27). The three cohort studies followed representative samples of participants stratified by sex and age, which were drawn in comparable ways from their respective regions (28) (GHS: *N* = 15 010 respondents, 35–74 years; KORA: *N* = 18 079 respondents, 25–74 years; SHIP: *N* = 12 324 respondents, 20–79 years). The three German regions differ regarding socioeconomic and economic parameters: Unemployment rates are highest, discretionary incomes, and life expectancy lowest in Greifswald (SHIP). As the eastern and western states of Germany have evolved in different political and economical systems following the 2nd World War, these regions show pronounced differences regarding full-time employment rates among mothers resulting in different gender pay gaps (28).

These three cohorts also include mental health variables and are therefore particularly suitable to derive knowledge on sex, mental health and their relationships to physical health. The GESA consortium (GEnder Sensitive Analyses of mental health trajectories and implications for prevention) has recently been established to enable future conjoint analyses (28). The purpose of this systematic review is to analyse published findings from the three cohorts and to identify sex-specific prevalence rates of mental disorders and to identify risk and protective factors and regional effects.

## Methods

Published studies were identified through searches of the databases PubMed and Web of Science. We included studies from initiation of each cohort until June 2019. KORA started in 1996 (25) following its precursor MONICA which had started in 1984, SHIP started in 1996 (26) and GHS started in 2007 (27). Keyword, title, authors and abstract information were used. The search terms were “*KORA*,” “*Cooperative Health Research in the Augsburg Region*,” “*SHIP*,” “*Study of Health in Pomerania*,” “*GHS*” or “*Gutenberg Health Study*” combined with “*mental health*,” “*sex*,” or “*gender*.” English and German publications were considered. Unpublished papers, abstracts, dissertations and book chapters were not included. The search took place from April until June 2019.

To decide whether an article fulfilled the criteria, the first rough selection was made by inspecting the title and abstract of the article. To identify relevant studies, the following inclusion criteria were used:

1) studies using KORA, SHIP or GHS data;
2) cross-sectional and longitudinal/prospective studies addressing mental health;
3) studies containing sex, respectively gender.

**Figure 1:**
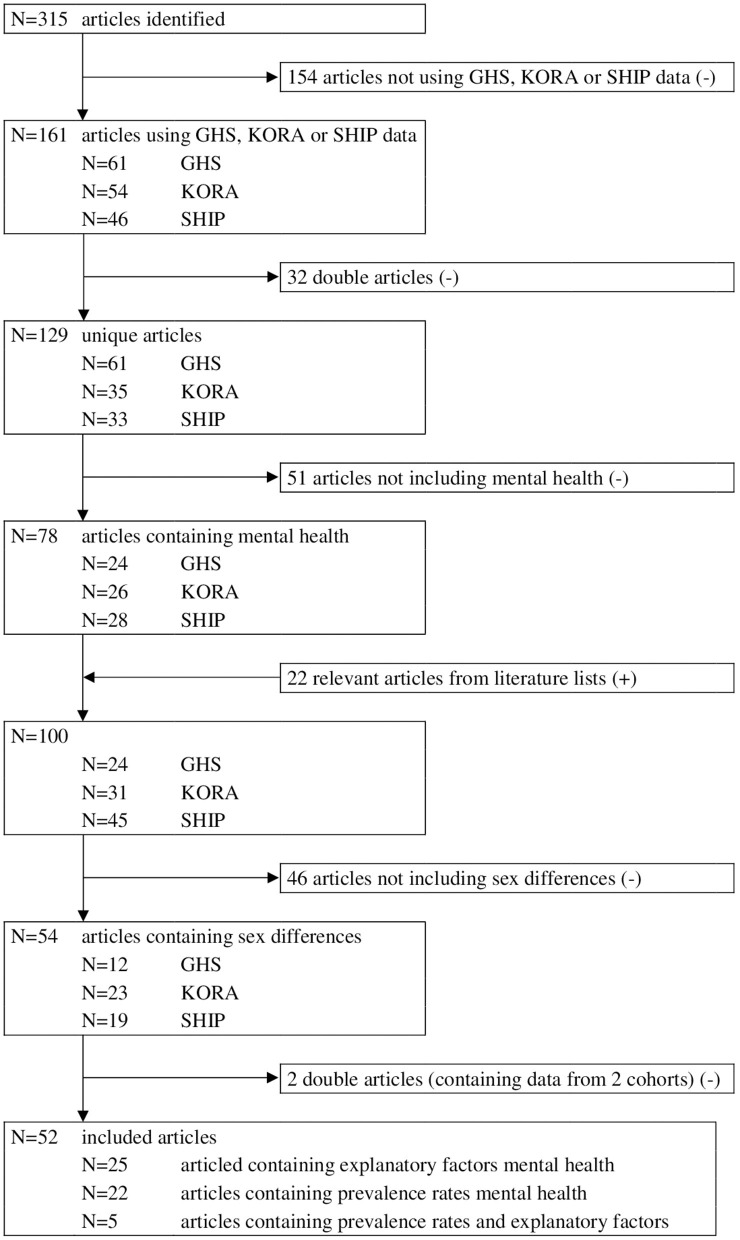
Overview of identified, screened and included articles.

Furthermore, it was required that the articles either (1) contained descriptive statistics on mental health variables including statistical tests that probed for differences between women and men, or (2) tested factors related to mental health with separate analyses for women and men, respectively analyses with an interaction term for sex. We only selected papers that included mental health as a dependent variable. This first group of articles was used to describe prevalence rates of mental health for women and men. The second group of articles was analyzed to describe explanatory factors for mental health between women and men. Of the selected articles the literature list was meticulously examined and further relevant articles were selected. Articles including analyses with mental health as dependent variable, but only including sex as a control factor, were not taken into account. Articles containing descriptive statistics for mental health aspects, but without differences between women and men (statistically tested) were excluded. Lastly, we excluded methodological papers. The selection procedure is displayed in detail in Figure 1.

Mental health is a broad concept. In this article, we were guided by the definition of WHO including mental, physical and social well-being, not just the absence of mental disorders (29). Similarly, Galderisi et al. (30) interpreted mental health as a dynamic state of well-being changing through lifetime and depending on life events. Therefore, not only mental disorders, but also mental health aspects such as well-being, somatic symptoms, loneliness and resilience were taken into account in this review.

## Results

Our search strategy has identified 52 articles reporting GHS, KORA or SHIP data on mental health and sex differences. We found articles reporting prevalence rates of mental health [*N* = 22], explanatory factors for mental health [*N* = 25] and articles reporting both [*N* = 5].

Articles assessing gender differences based on gender identity and gender relations were not found, but some articles included variables indicating gender aspects related to gender roles and institutionalized gender. Previous research indicated gender differences in power, responsibilities and in dimensions of the self (4), contributing e.g., to lower income of women, more responsibilities in childcare and more domestic work. In another previous study a gender index based on secondary data specified taking care of children, being unemployed, working few hours and lower education as indicators for feminine gender roles (31). Such socioeconomic and family-related factors were included as possible gender factors.

### Prevalence of Mental Health Complaints

The articles reported a broad range of prevalence rates of mental health indicators for women and men. The articles are consistent in their findings, but most prevalence rates were only reported in one or two of the three cohorts, based on specific diagnostic instruments. The results are listed in Table 1.

**Table 1:** Results prevalence rates mental health for women and men.

**References**	**Study**	***N***	**Mental health construct**	**Measurement scale, cut-off value (range)**	**Total**	**Women^1^**	**Men^1^**	***p*-value^2^**
Grabe et al. (32)	SHIP	3,300	Depression	CID-S^6^: two items, ≥1 (0–2)	4,310	807 (36.8%)	507 (23.9%)	***p*** **<** **0.05**
			Anxiety	CID-S^6^: five items, ≥1 (0–5)	4,310	1,223 (55.8%)	823 (38.9%)	***p*** **<** **0.05**
			Somatization	CID-S^6^: one item, ≥1 (0–1)	4,310	384 (17.5%)	308 (14.5%)	***p*** **<** **0.05**
Grabe et al. (33)	SHIP	3,045	Traumatic event	PTSD module of SCID^7^, ≥1^7a^	3,045	830 (52.8%)	833 (56.5%)	***p*** **<** **0.05**
			PTSD^3^	PTSD module of SCID^7^, ≥6 (0–17)^7b^	1,663	43 (5.2%)	24 (2.9%)	***p*** **<** **0.05**
Spitzer et al. (34)	SHIP	3,171	PTSD^3^	PTSD module of SCID^7^, ≥1 (0–17)^7c^	3,171	42 (2.5%)	20 (1.3%)	***p*** **≤** **0.007**
Michal et al. (35)	GHS	4,912	Suicidal ideation	Item PHQ-9^8^, ≥1 (0–3)	4,912	223 (9.2%)	151 (6%)	***p*** **<** **0.001**
Appel et al. (36)	SHIP	2,157	Depressive symptoms	BDI-II^9^, continues (0–63)	2,157	7.0 ± 7.9%	5.7 ± 6.3	***p*** **<** **0.001**
			MDD^4^	M-CIDI^10^	2,157	253 (22.3%)	117 (11.5%)	***p*** **<** **0.001**
Michal et al. (37)	GHS	4,900	Depersonalization	CDS-2^11^, ≥3 (0–6)	4,900	20 (0.8%)	21 (0.8%)	*p* = 0.96
Spitzer et al. (38)	SHIP	1,772	Mental health problems	CID-S^6^, ≥1 (0–12)	1,772	372 (40.9%)	261 (30.2%)	***p*** **≤** **0.001**
Wiltink et al. (39)	GHS	5,000	Mental distress^5^	PHQ-9^8^, ≥10 (0–27); PHQ panic module^12^, ≥2 (0–4); Mini-Spin^13^, ≥6 (0–12); GAD^14^-2, ≥3 (0–6)	4,753	453 (19.4%)	304 (12.6%)	***p*** **≤** **0.001**
Beutel et al. (40)	GHS	4,928	Type D personality	DS14^15^, ≥10 (0–28)	4,928	561 (23.1%)	534 (21.3%)	*p* = 0.13
Grabe et al. (41)	SHIP	2,035	Depressive symptoms	BDI-II^9^, continues outcome (0–63)	2,035	7.0 ± 7.9	5.7 ± 6.5	***p*** **<** **0.001**
Grabe et al. (42)	SHIP	1,974	Depressive symptoms	BDI-II^9^, continues outcome (0–63)	1,974	7.0 ± 7.8	5.6 ± 6.2	***p*** **<** **0.001**
Häfner et al. (43)	KORA	1,369	Sleep disturbances, no depressed mood	USI^16^, ≥3 (1–6); DEEX^17^, ≥12 women; ≥10 men (0–24)	1,369	157 (22.1%)	95 (14.4%)	***p*** **<** **0.01**
Perna et al. (44)	KORA	3,347	Resilience	RS-11^18^, upper third	3,347	536 (30.5%)	501 (31.5%)	*p* = 0.173
Ladwig et al. (45)	KORA	3,000	Full PTSD^3^	PDS and IES^19^	3,000	32 (2.1%)	19 (1.3%)	***p*** **<** **0.001**
Michal et al. (46)	GHS	4,937	Depression	PHQ-9^8^, ≥10 (0–27)	4,937	212 (8.7%)	145 (5.8%)	***p*** **<** **0.001**
Michal et al. (47)	GHS	9,751	Severe sleep problems	Item PHQ-9^8^, ≥2 (0–3)	9,751	548 (11.4%)	354 (7.2%)	***p*** **<** **0.001**
Schneider et al. (48)	KORA	12,888	Commiting suicide	Death certificates	12,898	18 (0.3%)	30 (0.5%)	***p*** **=** **0.001**
Zebhauser et al. (49)	KORA	1,022	Mild/moderate depression	Geriatric Depression Scale-15^20^, ≥4 (0–15)	1,058	87 (17.2%)	60 (10.9%)	***p*** **=** **0.011**
			Anxiety	GAD-7^14^, ≥10 (0–21)	1,005	53 (10.6%)	24 (4.8%)	***p*** **=** **0.005**
			Low life satisfaction	Item Satisfaction With Life Scale^21^, ≤4 (0–10)	1,022	32 (6.3%)	24 (4.7%)	*p* = 0.24
			Low resilience	RS-11^18^, lowest two thirds	993	356 (71.8%)	333 (67%)	***p*** **=** **0.10**
Zebhauser et al. (50)	KORA	346	Living alone and loneliness	Item living alone (dichotom); UCLA- Loneliness-Scale-12^22^, ≥21 (0–36)	346	76 (29.6%)	29 (32.6%)	*p* = 0.59
Beutel et al. (51)	GHS	14,661	Severe loneliness	Item frequency alone, =4 (0–4)	14,413	165 (2.3%)	83 (1.2%)	***p*** **<** **0.001**
Goltz et al. (52)	SHIP	3,926	Depression and obesity	PHQ-9^8^, ≥10 (0–27); WHR^23^, > 0.85 females; > 1.0 males	3,926	77 (3.8%)	25 (1.3%)	***p*** **<** **0.010**
Rose et al. (53)	GHS	7,930	Fatigue	PBS^24^, ≥50 (0–100)	7,948	1,266 (35.8%)	918 (20.9%)	***p*** **<** **0.001**
Atasoy et al. (54)	KORA	9,340	Depressed mood	DEEX^17^, ≥12 women; ≥10 men (0–24)	9,340	1,702 (37.6%)	1,814 (37.7%)	*p* = 0.96
Beutel et al. (55)	GHS	12,061	New onset of depression	PHQ-9^8^, ≥10 (0–27)	10,036	232 (5%)	205 (3.8%)	***p*** **=** **0.003**
König et al. (56)	SHIP	2,265	Lifetime MDD^4^	M-CIDI^10^	2,265	267 (22.5%)	119 (11%)	***p*** **<** **0.001**
Beutel et al. (57)	GHS	7,974	Somatic symptom load	PHQ-12^25^, ≥4 (0–24)	7,974	1,121 (28.8%)	1,073 (16.1%)	***p*** **<** **0.001**
Schlax et al. (58)	GHS	12,484	Depressive symptoms	PHQ-2^8^, ≥2 (0–6)	12,484	1,598 (26.4%)	1,281 (19.9%)	***p*** **<** **0.001**

1 *Number and percentage or mean and standard deviation*.

2 *Bold printed values significant*.

3 *Post-traumatic stress disorder*.

4 *Major depressive disorder*.

5 *Depression, panic, social anxiety or generalized anxiety*.

6 *Composite International Diagnostic Screener (CID-S): 12 yes/no questions addressing different diagnostic domains*.

7 *PTSD module of the Structured Clinical Interview for DSM-IV (SCID) measures experience of traumatic event and PTSD symptoms, including criterion A2 (experiencing high distress during/after event), criterion B (five re-experiencing symptoms), criterion C (seven avoidance symptoms), and criterion D (five arousal symptoms)*.

7a *Experiencing more than one traumatic event varying from combat or war zone experience to rape or abuse and illness*.

7b *Experiencing one re-experience (0–5), three avoidance (0–7) and two hyperarousal (0–5) symptoms*.

7c *Experiencing one re-experience (0–5) [no avoidance (0–7) or hyperarousal (0–5)] symptoms*.

8 *Patient Health Questionnaire 2 or 9 (PHQ-2 or PHQ-9) screens, diagnoses, monitors and measures severity of depression with two or nine items (4-point-Likert rating scale, 0–3)*.

9 *Beck Depression Inventory-II (BDI-II) measures presence and severity of depressive symptoms with 21 items (4-point-Likert rating scale, 0–3)*.

10 *Munich-Composite International Diagnostic Interview (M-CIDI) assesses psychiatric disorders over the lifespan according to DSM-IV criteria*.

11 *Brief Cambridge Depersonalization Scale (CDS-2) measures frequency and duration of depersonalization symptoms with two items (4-point-Likert rating scale, 0–3)*.

12 *Brief PHQ Panic Module screens panic disorders with four dichotomous items*.

13 *Mini-Social Phobia Inventory (Mini-Spin) detects social anxiety with three items (5-point-Likert rating scale, 0–4)*.

14 *Generalized Anxiety Disorder 2 or 7 (GAD-2 or GAD-7) screens for anxiety disorders with 2 or 7 items (4-point-Likert rating scale, 0–3)*.

15 *Type D scale (DS14) comprises two reliable subscales with seven items each for negative affectivity and social inhibition (5-point-Likert rating scale, 0–4)*.

16 *Adapted version of Uppsala Sleep Inventory (USI) measures initiating and maintaining sleep with two items (3-point-Likert rating scale, 1–3)*.

17 *DEpression and EXhaustion subscale (DEEX scale) measures presence and severity of depression and anxiety with eight items (4-point-Likert rating scale, 0–3). Sex-specific cut-off points were applied (≥12 for women, and ≥10 for men)*.

18 *Resilience scale 11 (RS-11) with 11 items (7-point-Likert scale, 1–7); highest one third indicates resilience, lowest two thirds indicates low resilience (thirds based on data)*.

19 *Post-traumatic Diagnostic Scale (PDS) and impact of event scale (IES) indicate, respectively experience of a traumatic event (criteria A, 11 events) and symptoms of PTSD (criteria B, C, and D; re-experiencing, avoidance and arousal). Exposure to one traumatic event and 1 re-experience, one avoidance and 1 hyperarousal symptom indicate full PTSD*.

20 *Geriatric Depression Scale-15 measures depression among elderly with 15 dichotomous items*.

21 *Satisfaction With Life Scale: one item measuring satisfaction with life on a 0–10 rating scale*.

22 *UCLA loneliness scale-12 measures loneliness with 12 items with a 4-point-Likert rating scale (0–3)*.

23 *Waist-to-hip ratio (WHR) is calculated by dividing the waist circumference through the hip circumference and therefore can adapt divergent values*.

24 *Personal Burnout Scale (PBS) measures physical and mental exhaustion with six items with a 5-point-Likert rating scale (1–5). Data were transformed to a metric scale (1 = 0; 2 = 25; 3 = 50; 4 = 75; 5 = 100)*.

25 *Patient Health Questionnaire 12 (PHQ-12) contains the somatic symptom module of the Patient Health Questionnaire-15 (PHQ-15): 12 items with a three-point-Likert rating scale (0–2)*.

In general, women reported more mental health problems than men (38, 39). Regardless of the measurement scale, depression or depressive symptoms were reported more often by women than by men (32, 36, 41, 42, 46, 58). This also applied to new onset of depression (55) and additionally to specific subgroups, e.g., elderly (49). Major depressive disorders were more frequently present among women compared to men (36, 56). However, when the depression and exhaustion subscale was used, no difference between women and men in reporting depressed mood was found (54). Depression combined with obesity was more often seen in women (52). Depending on operationalization, depression or depressed mood in women ranged from 8.7% (GHS) to 36.8% (SHIP) and in men from 5.8% (GHS) to 37.7% (KORA). Suicidal ideation was more often present in women (9.2% GHS) compared to men (6.0% GHS) (35). However, the risk of committing suicide was higher for men (0.5% KORA) than for women (0.3% KORA) (48). Lastly, anxiety was more often present among women (55.8% SHIP) compared to men (38.9% SHIP) (32), this also applied to elderly women (10.6% KORA) and men (4.8% KORA) (49).

Women reported more somatic complaints compared to men (32, 57). This varied from 17.5% (SHIP) to 28.8% (GHS) for women and from 14.5% (SHIP) to 16.1% (GHS) for men. Sleeping problems (47) and fatigue (53) were more often reported by women. In addition, the combination of sleep disturbances and depressed mood was more frequent among women (43). Men reported more traumatic life events than women (33), whereas women developed more often (lifetime) post-traumatic stress disorder (PTSD) after experiencing such an event (33, 34, 45). In women, PTSD ranged from 2.1% (KORA) to 5.2% (SHIP). In men, PTSD ranged from 1.3% (KORA and SHIP) to 2.9% (SHIP). Loneliness was also more frequently prevalent in women than in men (51). For women in the age groups 35–44 years and 55–64 years, living alone was strongly associated with loneliness (51). For elderly who lived alone, no difference was found for loneliness between women and men (50). Other indicators of mental health, such as presence of Type D disorder (40) and depersonalization-derealization (37) did also not differ between women and men. For elderly, no difference between women and men was found regarding resilience (44, 49) and life satisfaction (49).

### Factors Associated With Mental Health

A broad range of factors was associated with mental health. We categorized the results in five categories: (1) social and gender-related factors, (2) lifestyle, (3) interplay of physical and mental health, (4) body mass index (BMI) and diabetes and (5) genetic and biological factors. An overview of the results can be found in Table 2. The main findings are described below.

**Table 2:** Explanatory factors mental health for women and men from the three cohorts.

**References**	**Design^a^**	***N***	**Wave^b^**	**Main results^e^**
Grabe et al. (59)	CS	976	SHIP-0 (non-random)	° Low social support associated with high mental and physical distress (f). ° Chronic diseases associated with high mental and physical distress (f, m). °*S/s*-genotype and *s/l*-genotype on serotonin transporter gene (*5-HT*) and unemployment related to higher mental and physical distress compared to l/l genotype (f). °*S/s*-genotype and *s/l*-genotype on the *5-HT* and chronic diseases related to higher mental and physical distress compared to *l/l* genotype (f).
von Lengerke et al. (60)	CS	947	KORA-Survey-2000	° No differences between f and m in associations between different weight groups and mental health-related quality of life (HRQL).
Grabe et al. (61)	CS	1,059	SHIP-0 (subjects aged ≥ 60)	° More years in school, respectively 12 (f) or 10 (f, m) related to better mental status for older people. ° For older people, higher income, respectively in the 3rd quintile (m) or in the 4th and 5th quintile (f, m) related to better mental status. ° Five or more children associated with worse mental status (older f, m). ° A smaller number of teeth negatively associated with mental status (older f).
Lucht et al. (62)	CS	406	SHIP-I	° Oxytocin receptor gene (*OXTR*) *rs53576 A/A* associated with less positive affect (m).
Wiczinski et al. (63)	CS	2,732	KORA F3	° Normal weight more often present in younger and higher educated people (f, m). ° Not living with partner related to a higher Body Mass Index (BMI) (m). ° Statutory health insurance associated with a higher BMI (f). ° Higher BMI (direction obese) associated with lower physical HRQL (f, m). ° Interaction between social support and BMI on physical HRQL (m). ° No association between BMI and mental HRQL (f, m).
Grabe et al. (64)	CS	1,638	SHIP-LEGEND^c^	° No interaction effect of emotional neglect and Corticotropin-releasing hormone receptor (*CRHR1*) gene on depression (f, m). ° No interaction effect of abuse and *CRHR1* gene on depression (f, m). ° For male interaction effect of physical neglect and *CRHR1* gene on depression.
Ladwig et al. (65)	CS	3,079	KORA F3	° Low net income, physical inactivity, high level of somatic complaints and pulmonary disease symptoms associated with suicidal ideation (SID) (f, m). ° Unemployment, living alone, cigarette smoking, medium level of somatic complaints, myocardial infarction associated with SID (m).
Ladwig et al. (66)	CS	11,895	MONICA S1 MONICA S2 MONICA S3	° High cholesterol, obesity, hypertension, low alcohol consumption and diabetes mellitus related to excessive symptom reporting (ESR) (f, m). ° No partner (f), lower educational level and higher unemployment (f, m) associated with ESR. ° Diseases (metabolic syndrome, circulatory diseases, bronchial diseases, cancer and other diseases) related to ESR (f, m). ° Regular sleep disturbances, not a good health perception and psychological symptoms related to ESR (f, m).
Grabe et al. (67)	CS	4,308	SHIP-0	° Confirmation of findings of the original paper of Grabe et al. (59) with an updated sample.
Häfner et al. (68)	CS	1,547	MONICA S1 MONICA S2 MONICA S3	° Depression associated with social isolation (f), physical inactivity (m) and lower BMI (m). ° Inflammatory marker high-sensitivity C-reactive protein (*hs-CRP*) not associated with depression or social isolation. °*Hs-CRP* levels higher for people with depression and social isolation (m). ° Inflammatory marker interleukin-6 (IL-6) associated with social isolation (m). ° IL-6 levels higher for people with depression and social isolation (m).
Häfner et al. (69)	CS	1,229	MONICA S1 MONICA S2 MONICA S3	° Higher C-reactive protein (*CRP*) levels associated with social isolation and (no) depression (m). ° Higher leptin levels associated with social isolation/integration and depression (m). ° Smoking (m) and no partner (f) associated with social isolation (regardless of having depression). ° No partner (m) and higher age (f) associated with social isolation without depression. ° Sleeping disorder, high somatic complaints and negative self-perceived health associated with depression (regardless social isolation or integration) (f, m). ° Physically inactivity related to social isolation (regardless of having depression) (f, m) and to social integration and having depression (m).
Schunk et al. (70)	CS	9,579	KORA S4 SHIP-0	° Type 2 diabetes related to lower score on mental HRQL (f). ° No differences in associations between no Type 2 diabetes and mental HRQL (f, m).
Grabe et al. (41)	CS	2,035	SHIP-LEGEND^c^	° A three way interaction between (1) *brain-derived neurotrophic factor (BDNF) Met allele*, (2) *biallelic/triallelic 5-HTTLPR ss-allele* and (3) non/mild childhood abuse influenced depression (f).
Häfner et al. (43)	CS	1,369	MONICA S1 MONICA S2 MONICA S3	° No associations between leptin levels and depressed mood or sleep disturbances (f, m). ° Associations between leptin levels and an interaction between depressed mood and sleep disturbances (f). ° Higher leptin levels related to depressed mood and sleep disturbances in normal weight people (f). ° For people with obesity and elevated leptin levels no associations between higher leptin levels and depressed mood and sleep disturbances (f).
Lukaschek et al. (71)	CS	3,080	KORA S4	° Combat or war zone (f, m), non-sexual assaults by known assailants (f, m), experience of interpersonal conflict (m), sexual assaults by known assailant (m) and life threatening diseases (f) important factors for development of full Post-Traumatic Stress Disorder (PTSD). ° For men experience of interpersonal conflict (f, m), life threatening illness (f, m), having a serious accident (m) and sexual or non-sexual assaults by known assailants (f) important factors for development of partial PTSD.
Schomerus et al. (72)	CS	395	SHIP-0 SHIP-I SHIP-LEGEND^c^	° No differences between f and m in the association between childhood abuse, personality traits and resilience and help-seeking for depression.
Wiltink et al. (73)	CS	5,000	GHS BL	° Obesity measure waist-to-hip ratio (WHR) positively related with depression and somatic-affective symptoms (m). ° Obesity measures BMI and waist-to-height ratio (WHtR) negatively related to cognitive-affective symptoms (f). ° Obesity measure waist circumference (WC) negatively associated with cognitive-affective symptoms (m).
Emeny et al. (74)	CS	985	KORA-AGE^d^	° Higher levels of insulin-like growth factor binding protein-3 (IGFBP-3) associated with higher well-being (elderly f). ° Higher insulin-like-growth factor 1 (IGF-I) levels associated with more depression (elderly f). ° No relation between IGFBP-3 and depression (elderly f, m). ° Active elderly f with the highest IGF-I and IGFBP-3 levels higher well-being than inactive elderly f with similar IGF-I levels and IGFBP-3 levels.
Hunger et al. (75)	CS & LS	1,046	KORA S4 KORA F4	° People of 55 years and older with prediabetes at baseline and diabetes at follow-up scored lower on mental HRQL than people with persistent prediabetes (m). ° People of 55 years and older with normal glucose tolerance (NGT) at baseline and prediabetes at follow-up scored lower on mental HRQL than people with NGT at follow-up (f).
Klug et al. (76)	CS	969	KORA-AGE^d^	° For elderly people higher attachment (m) and lower loneliness (f, m) scores related to not having late life depression.
Laxy et al. (77)	LS	3,080	KORA S4 KORA F4	° Heavy weight gain associated with impairments in physical health (f, obese m) and improvements in mental health (f).
Sievers et al. (78)	CS & LS	4,079 (CS) 3,141 (LS)	SHIP-0 SHIP-I	° Low IGF-I levels (f) and high IGF-I levels (m) predict incidence of depressive disorders.
Zebhauser et al. (49)	CS	1,022	KORA-AGE^d^	° People older than 85 years experience more loneliness than people between 64 and 84 years (f, m). ° Elderly women (>85) experience more loneliness than elderly men. ° For older people associations between a small social network and loneliness (f, m). ° Older age related physical limitations mostly not associated with loneliness (f, m). ° Inactivity related to loneliness (elderly f). ° Depression and anxiety associated with loneliness (elderly f, m). ° More life satisfaction and stronger resilience associated with not being lonely (elderly f, m).
Baumeister et al. (79)	LS	4,228 (SHIP-0) 4,251 (SHIP-T)	SHIP-0 SHIP-Trend	° Relative increase of depressive symptoms between 1997–2001 and 2008–2012 stronger for men than for women. ° Strongest change of depressive symptoms between 1997–2001 and 2008–2012 in age group 50–64 years (f, m); smallest change in age group 20–34 years (f, m). ° No difference in prevalence of depressive symptoms between 2008–2012 and 1997–2001 (f, m).
Johar et al. (80)	CS	733	KORA-AGE^d^	° Lower morning to evening cortisol ratio associated with cognitive impairment (elderly m). ° Late evening cortisol measures and cortisol awakening response (CAR) not associated with cognitive impairment (elderly f, m).
Schunk et al. (81)	CS	846	KORA S4 SHIP-0	° Using oral diabetic medication, insulin diabetic medication or oral and insulin diabetic medication associated with lower mental HRQL (f).
Lukaschek et al. (82)	CS	3602	KORA-AGE^d^	° For elderly low income, anxiety, depression, sleeping problems, physical inactivity and multi-morbidity associated with low subjective well-being (f, m). ° Living alone associated with low subjective well-being (elderly f).
Rabel et al. (83)	CS & LS	3,080	KORA S4 KORA F4 KORA FF4	° Women with a higher BMI later in life less physical HRQL than men. ° Women with no or low physical activity later in life less mental HRQL than men.
Beutel et al. (55)	LS	12,061	GHS BL GHS FU 2	° Cancer, loneliness, social phobia and generalized anxiety risk factors for new onset of depression (f). ° Smoking and life events risk factors for new onset of depression (m). ° Social support associated with less new onset of depression (f, m).
Beutel et al. (57)	CS	7,974	GHS FU 2	° Lack of social support, adverse life events, depression, generalized anxiety, panic and social phobia strongest predictors for somatic symptoms (f, m). ° Loneliness predictor for somatic symptoms (f).

a *CS, cross-sectional study; LS, longitudinal study*.

b *SHIP-0 = 1997–2001, SHIP-I = 2002–2006, SHIP-LEGEND = 2007–2010, SHIP-Trend = 2008–2012, KORA S4 (1999–2001), KORA F3 = 2004–2005, KORA F4 (2006–2008), KORA FF4 (2013–2014), MONICA S1 = 1984–1985, MONICA S2 = 1989–1990, MONICA S3 = 1994–1995, GHS BL = 2007–2012, GHS FU 1 = 2009–2014, GHS FU 2 = 2012–2017*.

c *SHIP-LEGEND is an add on study, based on the SHIP-0 cohort*.

d *KORA-AGE includes MONICAS1, MONICAS2, MONICAS3 and KORA S4. Participants aged 65 years or older at the end of 2008 were selected*.

e *f, female; m, male*.

#### Social and Gender-Related Factors

The three cohorts have been drawn as local representative samples of the general population, stratified for sex, age, and urban-rural residence. In all cohorts, women had a lower income than men. Furthermore, men were more often married or living with a partner, whereas more elderly women were living alone. In addition, life expectancy differed between the regions; life expectancy was highest in the middle of Germany and lowest in the northeast of Germany. The demographic and socioeconomic differences between the cohorts influenced associations with mental health.

Social support and social isolation were often associated with mental health. Lack of social support was a strong predictor for somatic symptoms for both women and men (57). For older women and men, a smaller social network was associated with loneliness (49). For women, low social support was associated with higher mental and physical distress (59) and women without a partner reported more excessively somatic symptoms (66) and were more often socially isolated (69). The presence of social support turned out to be a protective factor against new onset of depression for both women and men (55). For men, not having a partner was associated with social isolation (69) and living alone was linked to suicidal ideation (65). Additionally, elderly women living alone reported lower subjective well-being (82). Depressed women were more likely to be socially isolated (68), and older women were also more often socially isolated, regardless of suffering from a depression (69). For women as well as men, adverse life events were strong predictors for somatic symptoms (57). For men, negative life events were a risk factor for new onset of depression (55) and associated with the development of PTSD (55). Experience of combat or war zone and non-sexual assaults by known assailants were risk factors for developing full PTSD (71) for women and men. For help seeking when suffering from depression, no differences were found between women and men (72).

With regard to gender-related factors, findings for women and men were similar. Social economic status predicted somatic symptoms for women and men (57). Low income had a negative effect on well-being (82) and was associated with suicidal ideation (65). Having five or more children was associated with a worse mental health status (61) and low educational level and unemployment were related to excessive symptom reporting (66). A difference was found in the association between unemployment and suicidal ideation, which was only present for men (65). No effects were found for social economic status on new onset of depression (55), educational level on well-being (82) and unemployment on somatic symptoms (57). Furthermore, no association between net income and status as homemaker were found (65). In general, educational attainment, income and employment did not have an effect on the increase of depressive symptoms over the last years (79).

#### Lifestyle

Smoking and physical inactivity are known factors to negatively influence physical and mental health. Smoking was a risk factor for new onset of depression, but only in men (55). Besides, smoking was associated with suicidal ideation (65) and with social isolation (69) for men. For physical activity, effects for women and men were found. For both women and men being physically inactive was related to social isolation and for men also to social integration and suffering from depression (69). Another study confirmed that depressed men were more likely to be physically inactive than women (68). For both women and men associations between physical inactivity and suicidal ideation were found (65). Women who were moderately active had a lower mental health-related quality of life (HRQL) score than men who were moderately active (83). For older women physical inactivity was associated with loneliness (49), whereas the association between physical inactivity and low subjective well-being was found for both elderly women and elderly men (82). Low alcohol consumption was associated with excessive symptom reporting for both women and men (66).

#### Interplay of Physical and Mental Health

The interplay between physical and mental health has been examined in many studies included in the current review. For both women and men chronic diseases were associated with worse mental and physical well-being (59). For women the experience of a life threatening disease was associated with the development of full and partial PTSD, for men it was only associated with partial PTSD (71). For women cancer, social phobia, generalized anxiety and loneliness were risk factors for new onset of depression (55). Sleeping disorder, somatic complaints and negative self-perceived health were associated with depression for women as well as for men (69). For elderly women and men absence of loneliness was a protective factor for depression later in life (76), for elderly men, stronger attachment was an additional a protective factor. Depression, generalized anxiety, panic and social phobia predicted somatic symptoms for women and men (57), for women somatic symptoms were also predicted by loneliness. Increased reporting of somatic symptoms was predicted by diseases in general (metabolic syndrome, circulatory diseases, bronchial diseases, cancer and other diseases) for both women and men (66) and associated with sleep disturbances, worse health perception and presence of psychological symptoms. High levels of somatic complains and pulmonary disease symptoms were associated with suicidal ideation for women and men (65), for men medium level of somatic complaints, myocardial infarction and stroke were additionally associated with suicidal ideation. For women as well as for men, depressive symptoms were associated with suicidal ideation (65).

#### Body Mass Index (BMI) and Diabetes

Obesity and diabetes are significant health threats in an aging population. When comparing women and men in the association between BMI and HRQL (physical and mental), women with a higher BMI reported lower physical HRQL later in life than men with a higher BMI (83). Another study found an association between high BMI and less physical HRQL for both women and men (63), but in men with strong social support, this association vanished. No association was found between BMI and mental HRQL for either women or men (63) and no differences were found between women and men (60, 83). Women with type 2 diabetes scored lower on mental HRQL compared to men with Type 2 diabetes (70), regardless of type of diabetic medication (81). Changes in the diabetes trajectory also influenced mental HRQL. Men of 55 years and older with prediabetes at baseline and diabetes at follow-up scored lower on mental HRQL than men with persistent prediabetes (75). Women with normal glucose tolerance at baseline and prediabetes at follow-up scored lower on mental HRQL compared to women who still had normal glucose tolerance at follow-up (75). For women and overweight men weight gain was associated with impaired physical health (77). For women, weight gain was associated with improvements in mental health (77). Men with lower BMI more often suffered from a depression (68) and lower BMI was associated with social isolation (69). In addition, diabetes mellitus and obesity were related to excessive somatic symptom reporting (66). Furthermore, using different indicators for obesity [e.g., waist-to-hip ratio (WHR) or BMI] led to different findings (73).

#### Genetic and Biological Factors

Genetic and biological factors may affect mental health status and the development of mental health disorders. For mental and physical distress, the *s/s*-genotype and the *s/l*-genotype on the serotonin transporter gene in combination with unemployment or chronic diseases was related to higher mental and physical distress for women (59, 67). Elderly women reported better well-being when higher levels of insulin-like growth factor binding protein-3 (IGFBP-3) were present. A discrepancy between women and men was found for insulin-like-growth factor 1 (IGF-I). For women, low IGF-I levels predicted the incidence of depressive disorders 5-years later, whereas for men high levels of IGF-I predicted future depression (78). In another study an association between IGF-I levels and current depression was found for elderly women, but contrary to the previous results; higher IGF-I levels were associated with more depression (74). Furthermore, for women lower cholesterol levels were associated with suffering from depression (69). Higher cholesterol levels were associated with increased symptom reporting of somatic symptoms and with hypertension for women and men (66). For men higher C-reactive protein (CRP) levels were associated with social isolation and the presence of depression (69). The inflammatory marker high-sensitivity C-reactive protein (hs-CRP) was not associated directly with either depression or social isolation, but for men with a depressed mood hs-CRP levels were higher compared to men without depressed mood and without being socially isolated (68). No associations between leptin levels and depressed mood or sleep disturbances were found for either women or men (43). However, in normal weight women, leptin levels were higher for women suffering from both depressed mood and sleep disturbances. Associations between cortisol levels and cognitive functioning depend on time of day measurement of cortisol levels (80). Associations between cortisol levels and loneliness were found in married elderly men, but not in married elderly women (84). A study focussing on the oxytocin receptor gene rs53576 A/A found an association between oxytocin receptor gene and less positive affect (strong excitement/emotion) for men (62). A three way interaction between *brain-derived neurotrophic factor: Met allele, biallelic 5-HTTLPR: ss-allele* and no/mild childhood abuse was found to influence depression for women (41). For men an interaction effect of physical neglect with Corticotropin-releasing hormone receptor 1 gene on depression was found (64).

## Discussion

In this systematic review, we compared prevalences and determinants of mental health indicators from three large, ongoing German cohorts. The benefits of combining three different cohorts are multiple. First, the cohorts represent the general population in three regions, namely KORA in the south, SHIP in the northeast and GHS in the middle of Germany. In these three regions, the composition of populations differs with respect to socioeconomic and regional characteristics. Therefore, the articles complement each other and the conclusions based on concurrent evidence are stronger. Secondly, the stratified random sample selection in all cohorts provides representative groups for the general adult population in the respective areas of Germany. The proportions of women and men in the cohorts are equal and broad ranges of age are covered. Repeated follow-ups provide the opportunity to study participants longitudinally and determine life-span developments. A third advantage of are the overlapping psychological, medical and laboratory assessments. Lastly, the overall number of participants from the three cohorts together is more than 40 000 (28).

Mental health plays a major role in the concurrent German cohort studies and sex differences are increasingly taken into account in analyses. This is underlined by the differences of prevalence rates of mental distress between women and men. In all cohorts, women reported worse mental health than men (38, 39, 85). Particularly, depression, depressed mood or symptoms were more often present in women (32, 36, 41, 42, 46, 52, 54, 56, 58). Additionally, as reported in one or two of the three cohorts, new onset of depression (55), anxiety (32), traumatic events and PTSD (33, 34, 45), suicidal ideation (35), sleeping problems and fatigue (47, 53), somatic complaints (32, 57) and loneliness (51) were more often reported by women, whereas the risk of committing suicide was stronger in men (48). A recent paper using SHIP data confirmed that women were more often affected by depressive, anxiety, obsessive-compulsive disorder, PTSD, somatoform and eating disorders (12-month and lifetime), whereas men indicated substance use more often (86). These results indicate similarities between cohorts and thus regions in sex-specific prevalence rates of mental health. Furthermore, these findings are consistent with current literature on prevalence rates of mental health in several countries. A meta-analysis on depression including 90 studies from 30 countries reported consistently higher prevalence rates of depression in women compared to men (87). In a review on social anxiety disorder including 14 studies from different countries, women were more often suffering from anxiety and reported elevated severity compared to men (88). A systematic review on suicidal behavior and suicide in Europe and America reported that men commit suicide more frequently, whereas women have more attempts and suicidal behavior (89). Furthermore women reported more often lifetime PTSD (90), somatoform diseases (91) fatigue (92, 93) and loneliness (94, 95). The higher prevalence of loneliness is especially present in older women and consistent across several European countries (96).

With regard to risk and protective factors for mental health, differences and similarities were found between women and men. For women loneliness, social isolation and low social support were risk factors for depression or mental distress (55, 59, 68, 69, 76). This finding was consistent in the three cohorts Further, for women, cancer, social phobia, generalized anxiety (55), sleeping disorders and negative self-perceived health (69) were associated with (new onset of) depression. For men myocardial infarction and stroke were associated with suicidal ideation (65). As men experience cardiovascular events at younger age (97), their consequences may lead to a high burden in daily life which can contribute to an increase of suicidal ideation. Additionally, for men, physical inactivity was associated with depression (68, 69) and smoking with new onset of depression (55), suicidal ideation (65) and social isolation (69). With regard to biological factors, for women, lower cholesterol levels were associated with depression (69) and higher leptin levels with depressed mood (43). For men, higher interleukin-6 levels and higher hs-CRP levels were associated with depression and social isolation (68), higher leptin levels predicted depression and social isolation (69). For other mental health outcomes, no differences were found between women and men. Chronic and life threatening diseases were associated with more mental and physical distress for women as well as for men in all three cohorts (59, 66, 67). Further, for both women and men associations were found between somatic complaints and depression (69) and suicidal ideation (65), life threatening disease and partial PTSD (71) and pulmonary disease and suicidal ideation (65). Additionally, depression, generalized anxiety, panic and social phobia were predictors of somatic symptoms for both sexes (57). Lastly, no differences between sexes were found in the association of BMI with mental HRQL (60, 63, 83).

Not all mental health aspects and possible risk and protective factors were examined in all cohorts, this makes it more complicated to draw conclusions about regional similarities and differences. The most consistent and detailed findings were found for depression. In all cohorts depression, depressed mood or symptoms of depression were more often present in women compared to men (32, 36, 41, 42, 46, 52, 54, 56, 58). This gender gap is known to become manifest already in early adolescence and declines in early adulthood, remaining stable afterwards (98). When controlling for demographic and socioeconomic background this gender gap persisted (99). However, many studies question whether men actually experience less depression or if depression among men just remains frequently undetected. In a study on men's attitude regarding depression, normative expectations regarding masculinity equated suffering from depression to a weakness (100). Conforming to such traditional masculine norms is problematic for help-seeking for depression (101). This may also contribute to the higher suicide rate in men. Men are less likely to seek treatment for depression and when they seek treatment, they are less likely to be diagnosed with major depressive disorder (97). This is in part due to different symptoms of depression in women and men. When examining depressed patients, men reported more anger attacks compared to women and scored higher on irritability and overreaction (102). When testing the Gotland Scale for Male Depression (103), irritability appeared to be the best indicator of male depression (104). Studies have also strong associations between alcohol abuse, depression and suicidal behavior interacting with biological factors in men (105) and alcohol consumption as a coping strategy for depression in men (106). Thus, depression in men is often masked by atypical symptoms, such as externalizing depression symptoms. For men, external risk factors for depression were also found in this review [e.g., smoking (55) and physical inactivity (44)], whereas the risk profile for women contained more internal factors [e.g., loneliness (55) and social isolation (68)].

Since gender was not measured directly in the studies reviewed, socioeconomic and family-related factors were examined as gender-related factors. In general, women were more often housewives (41), in maternity leave (41), or unemployed (59) compared to men. On average, men had a higher income and education (41) compared to women. Differences between women and men with regard to gender indicators and mental health were found for unemployment and suicidal ideation (65, 69). For men unemployment was positively associated with suicidal ideation. Other associations between gender indicators and mental health were found for both women and men. Income was negatively associated with suicidal ideation (65), positively with mental health status (61) and for elderly positively with subjective well-being (82). Education was negatively associated with excessive symptom reporting (66) and positively with mental health status (61). Lower social economic status predicted somatic symptoms (57) and having five or more children was associated with a worse mental health status (61). Lastly, unemployment was positively associated with excessive symptom reporting (66). Unfortunately, none of the articles examined a moderation or mediation effect of gender-related factors in the relation between sex and mental health. Therefore, it is not possible to determine the contributions of sex and gender. In general interactions between sex and socioeconomic position are understudied (19). A study in Brazil testing mediating effects of socioeconomic factors in the association between sex and mental health revealed that personal income and schooling at the age of 30 mediates the association between sex and depression, anxiety and common mental disorders (107), but not family income or maternal schooling. Testing socioeconomic and family-related factors in associations between sex and mental health in longitudinal studies could contribute notably to the understanding of gender aspects in mental health.

Only very recently, the binary assessment of sex has been supplemented by the third category, diverse sex. Therefore, this paper does not discuss gender diversity, although it is known that the mental health of the German LHBTI community (lesbian, gay, bisexual, transgender and intersex individuals) should be further examined (11). Mental health of the LHBTI community has been expected to differ from the overall population [e.g., depression and suicidal behavior (108, 109)] due to the heteronormative orientation of society (110, 111)”. Increasing social acceptance could lead to more possibilities to express one's gender identity and sexual orientation which could improve the health status of sexual minorities (11). A further limitation in this study is that mental health was measured by self-report questionnaires. Since the measurements tools used are reliable and valid, it will still be a good indication of reality. Different questionnaires were used to assess the same mental health construct. In order to overcome this last limitation, the GESA project, integrating data from these three cohorts to examine mental health, harmonizes data (28). Studying interactions between socioeconomic and vocational factors with sex (e.g., full employment of women in SHIP vs. the role of housewife in KORA and GHS) will help to identify differential impact of socioeconomic inequality.

Overall, this systematic review shows the differences and similarities in prevalence rates and determinants of mental health indicators between women and men, from genetic and biological factors to indicators of the social environment. While our picture is far from complete, gender gaps regarding income, education, or living in a relationship underline the need to differentiate mental health outcomes according to sex-related determinants of mental health. Differing risk and protective factors for mental health require a sex sensitive prevention approach. However, this review shows that current research on mental health still lacks a clear focus on gender roles and identities, which have recently been included in the Gutenberg Health Study. Future research should therefore illuminate sex differences and broader sex and gender diversity (112). Therefore, an increased focus on sex and gender in mental health research is of great importance.

## Author Contributions

This review was written as part of the GESA Project. All authors contributed to the development and refinement of the GESA consort. MB, EB, HB, HG, JK, K-HL, GS, and PW were grant holders. The idea for this paper came from DO and MB. DO performed the literature search, selection of paper and drafted, together with MB, the first version of the review, which was substantially revised by GS, AT, and EB. Improvements of HB, JK, K-HL, PW, and HG lead to the current version of this review.

## Conflict of Interest

The authors declare that the research was conducted in the absence of any commercial or financial relationships that could be construed as a potential conflict of interest.
